# *Convolvulus arvensis*: Antioxidant, Antibacterial, and Antifungal Properties of Chemically Profiled Essential Oils: An Approach against Nosocomial Infections

**DOI:** 10.3390/life12122138

**Published:** 2022-12-19

**Authors:** Ahmad Mohammad Salamatullah

**Affiliations:** Department of Food Science & Nutrition, College of Food and Agricultural Sciences, King Saud University, 11 P.O. Box 2460, Riyadh 11451, Saudi Arabia; asalamh@ksu.edu.sa

**Keywords:** medicinal plant, bacteria, fungi, infections, chemical analysis, volatile compounds

## Abstract

*Convolvulus arvensis* is a medicinal plant in the family Convolvulaceae, which is used in traditional phytotherapy. The objective of this work was conducted to valorize essential oils of *Convolvulus arvensis* (EOCA) in terms of chemical composition, antioxidant, and antibacterial properties. To achieve this objective, the chemical composition was performed by the use of GC-SM. Antioxidant power was effectuated by the use of DPPH, FRAP, and TAC assays. Evaluation of the antimicrobial power was conducted against clinically important pathogenic bacteria (*E. coli, K. pneumoniae, S. pneumoniae,* and *S. aureus*) and fungi (*A. niger, C. albicans,* and *A. flavus*) by the use of disc diffusion and minimum inhibitory concentrations (MICs) assays. The results showed that the yield of recovered EOs from *Convolvulus arvensis* was 0.34% of the total mass of leaves and mainly was rich in cuprenne (34%), thymol (20%), himachalene (16%), and longifolene (10%). Notably, EOCA exhibited important antioxidant effects, wherein IC_50_ (DPPH) and EC_50_ (FRAP) were determined to be 30 µg/mL and 120 µg/mL, respectively, while the total antioxidant power was determined to be 508.0 ± 6.0 µg EAA/mg. An important antibacterial effect was noted for EOCA as an excellent inhibition zone was recorded against all bacterial strains, particularly *K. pneumoniae* and *S. aureus* with 14.27 ± 0.42 and 21.35 ± 0.76 mm, respectively. Similarly, MICs of *K. pneumoniae* and *S. aureus* were 21.35 ± 0.76 mm and 28.62 ± 1.65 µg/mL, respectively. Noticeably, important antifungal activity was shown by EOCA against all fungal strains wherein the inhibition zone diameters against all fungal species ranged from 19.44 ± 1.10 to 20.41 ± 1.81 mm. Notably, MICs of EOCA against *F. oxysporum* and *C. albicans* were 18.65 ± 0.94 and 19.38 ± 0.58 g/mL, respectively. The outcome of the present work showed that EOs from *Convolvulus arvensis* can be used to conceptualize drugs to manage diseases relative to free radicals and infections.

## 1. Introduction

Since the beginning of human history, aboriginal people have relied heavily on wild plants to prevent nutritional and pathogen-related illnesses [1,2]. Plant-based products have been used by humans in a variety of contexts ever since prehistoric times. The intentional use of natural resources has been studied for over a thousand years [3,4]. Plants with medicinal properties have been used for treatment for a long time, and they continue to provide new tools to fight against a wide range of illnesses [3]. The presence of polyphenols, flavonoids, lignins, alkaloids, terpenoids, carotenoids, vitamins, etc., has been linked to the bioactive and antioxidant potentials of these plants according to previous research [5,6,7]. By preventing lipid oxidation, limiting rancidity, and eliminating harmful oxidative products, plants contribute to the preservation of the nutritional content of foods as well as its shelf life [8,9].

Plants continue to serve as the backbone of many current nutraceutical products. Plants produce several secondary metabolites as a defense strategy against environmental stress, or other factors such as insect infestations, wounds, and illnesses. Plants’ complex secondary metabolites have been used in medicine for a wide range of medicinal purposes for as long as medicine has existed [10,11]. Pharmacological investigations and drug realizations have relied heavily on medicinal plants for many decades. Many biologically active chemicals found in plants are produced via a process called secondary metabolism. These components are used in many therapeutic applications, either as active pharmaceutical ingredients or as prototypes for future medicinal molecules [12]. Edible and non-edible plants, as well as other natural sources, provide a wide variety of pharmaceuticals that may be found in nutraceuticals, nutritional supplements, and other forms of alternative medicine. Secondary metabolites are produced by plants, and these compounds have a wide range of medicinal pharmacological effects, including antibacterial, antioxidant, anticancer, and anti-inflammatory activity due to their unique chemical structures [13,14]. Medicinal plants are defined first and foremost as a comprehensive supply of bioactive chemicals that are used in the formulation of pharmaceuticals [15,16].

More attention has been paid to *Convolvulus* plant extracts and essential oils due to their biological properties, bioavailability, clinical efficacy, and safety [17]. The genus *Convolvulus* was found to be rich in flavonoids, steroids, terpenoids, carbohydrates, amino acids, anthraquinones [18], anthocyanidins, phenylpropanoids, coumarins, lignans, resins [17], tannins, saponins, alkaloids, lipids, and fatty acids [19,20]. Many species of the genus Convolvulus have been discovered to have medicinal characteristics, including those that may reduce or eliminate symptoms of major illnesses including fever, memory loss, sleeplessness, heart disease, hair loss [21], urogenital disorders, drastic purgative, gastrointestinal irritation, diarrhea, animal stings, congestion, edema, gaseous distended intestine, and hemorrhages of many organs [22].

Several pharmacological activities have been recorded for *C. arvensis* such as anticancer, food preservative, antispasmodic, antiproliferative, and cytotoxic activities [19]. This research work was conducted to investigate the chemical composition and antioxidant, antibacterial, and antifungal effects of *C. arvensis.*

## 2. Materials and Methods

### 2.1. Chemicals

2,3,5-triphenyl tetrazolium chloride (TTC), ammonium molybdate, sodium phosphate, quercetin, iron III chloride (FeCL3), and homologous series of *n*-alkanes (C8-C20) used in this investigation were all bought from Sigma-Aldrich (St. Louis, MO, USA), while culture medium, antibiotics, and (K3Fe(CN)6) were purchased from COGELAB (Kenitra, Morocco).

### 2.2. Extraction of Essential Oils

*Convolvulus arvensis* was collected in a desert area (31.285406, −4.243824) in 2022. Next, the plant was subjected to identification by a botanist and then deposited in the herbarium of the University (11/SaudK-08). The leaves were dried by use of an oven set to 40 °C for 4 days before being ground into fine powder by use of an electric mixer. Using a Clevenger apparatus, 100.00 g of the plant powder underwent hydro distillation for 3 h at 120 °C. Afterward, the obtained essential oils were kept in stained bottles at 4 °C for further pharmacological tests.

### 2.3. Phytochemical Analysis of EOCA

EOCA was analyzed by the use of a Thermo Fischer capillary gas chromatograph directly coupled to the mass spectrometer system (model GC ULTRA S/N 20062969; Polaris QS/N 210729), using an HP-5MS nonpolar fused silica capillary column (60 m × 0.32 mm, 0.25 μm film thickness) in addition to GC-FID (flame ionization detector) according to conditions detailed in previous work [23].

### 2.4. Antioxidant Potential

#### 2.4.1. DPPH Test

Shortly, 100 μL of various concentrations (0.001–1 mg/mL) of EOCA was mixed with 1.40 mL of 0.1 mmol DPPH solution [24,25]. Afterward, the mixtures were vortexed and incubated in darkness at room temperature for 31 min. Subsequently, the absorbance was read by use of a spectrophotometer at 517 nm against a blank with methanol only. Quercetin as a reference antioxidant was prepared under the same conditions as EOCA. The percentage of inhibition (I%) of DPPH free radical was calculated according to the formula:

I (%) = (Ab − As/Ab) × 100(1)

where As is the absorbance of the negative control and As is the absorbance of the test sample.

#### 2.4.2. FRAP Test

According to the method developed by Oyaizu, the iron-reducing capacity of EOCA was evaluated [26]. In summary, 200 μL of various concentrations of EOCA (0.001–1 mg/mL) were combined with 500.00 mL of phosphate buffer solution (0.2 M; pH 6.6) and 500 mL of 1% (K3Fe (CN)6). After incubating the mixture at 50 °C for 30 min, 500 mL of C2HCl3O2 (10%) was added to the reaction medium before centrifugation at 3000.00 rpm for ten minutes. Afterward, 500 mL of the supernatant from each concentration was mixed with 500 mL of distilled water and 100 mL FeCl3 (0.1%). The absorbance was measured at 700 nm using a spectrophotometer against a blank with methanol. Quercetin was used as a standard with absorbance measured under the same conditions as EOCA. The results were generated as EC50 (µg/mL), and the concentration of EOCA corresponding to 0.5 of the absorbance (EC50) was calculated by plotting the absorbance versus the concentration of EOCV.

#### 2.4.3. TAC Test

Briefly, 25 microliters of EOCA, one milliliter of a reagent solution (0.6 mol/L of sulfuric acid), 28 mmol/L of sodium phosphate, and 4 mmol/L of ammonium molybdate were combined before being used for dosing. Thereafter, the solution underwent a 90 min incubation period at 95 °C before being cooled to room temperature. At 695 nm, the absorbance was measured. Ascorbic acid was the reference standard and results were given in µg AAE/mg [27].

### 2.5. Evaluation of the Antimicrobial Potentiality of EOCA

The antibacterial power of EOCA was evaluated against bacterial strains, namely *E. coli*, *K. pneumoniae*, *S. pneumoniae,* and *S. aureus*. Meanwhile, antifungal power was evaluated against fungal strains, namely *A. niger*, *C. albicans*, *A. flavus*, and *F. oxysporum* by use of disk diffusion on solid medium and microdilution assays

#### 2.5.1. Antimicrobial Activity of EOCA by Disk Diffusion Method

The antimicrobial potential of EOCA was determined by the disk diffusion method according to the experiment of Gary et al. (1980). Each strain culture to be studied was streaked on Petri dishes containing Mueller–Hinton Agar (MHA) culture medium, which was prepared by dissolving 40 g of the powder in 1000 mL of distilled water. After autoclaving at 120 °C for 15 min, the medium was poured into sterile Petri dishes. The optical density of the suspensions was checked by use of a UV-Visible spectrophotometer at 625 nm and adjusted to between 0.08 and 0.1 nm, corresponding to suspensions containing 10^7^ to 10^8^ CFU/mL. Next, 1 mL of the microbial inoculum (10^8^ CFU/mL) was deposited on the surface of the culture medium (MHA) of each Petri dish. After 5 min of impregnation, the excess inoculum was removed by aspiration. Filter paper disks (6 mm in diameter) loaded with 15 μL of EOCA were placed in the inoculated Petri dishes. Then, the dishes were incubated at 37 °C for 18 h. Negative (15 µL of 0.2% agar) and positive controls (Kanamycin, Fluconazole, and Oxacillin of 0.02 mg/disc) were used under the same conditions, and after incubation, the inhibitory diameter was measured [27,28,29].

#### 2.5.2. Antimicrobial Activity of EOCA by Use of Microdilution Method

The objective of this method was to determine the minimum inhibitory concentration (MIC) from a concentration range of EOCA. The MIC of the EOs was determined using 96-well plates, according to the method reported elsewhere (NCCLS, 1999). The bacterial suspension was prepared in sterile physiological water with a concentration of 10^8^ CFU/mL. Then, 50 μL are taken and placed in a tube containing 5 mL of sterile physiological water (10^6^ CFU/mL). Next, 50 μL of MH broth was placed in the wells, and then various concentrations of tested EOs were placed in each microwell at a volume of 50 μL, to which 50 μL of the bacterial suspension was added. Finally, the plates were incubated for 18 h at 37 °C. Bacterial and fungal growth was visualized by adding 20 μL of the aqueous 2,3,5-triphenyl tetrazolium chloride (TTC) solution (1%) to each well, with additional incubation for 1 h. TTC is colorless, turning red in the presence of growing bacteria. MIC corresponds to the well with the lowest concentration of EO for which the mixture did not produce a red color. This is the lowest concentration of EO inhibiting visible bacterial growth [30].

### 2.6. Statistical Analysis

The results of this study were given using the means as well as the standard deviations of tests performed three times. The ANOVA and Tukey’s HSD test, as post hoc analysis of variance tests, were used to manage the multiple comparisons that were conducted. At *p* less than 0.05, a difference was considered significant.

## 3. Result and Discussion

### 3.1. Chemical Composition

**Figure 1:**
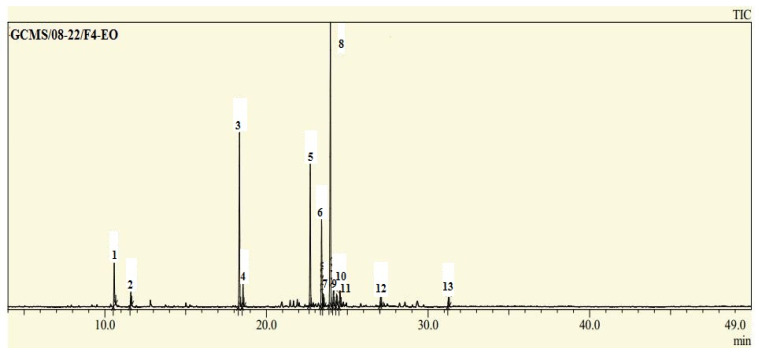
Gas chromatographic analysis (GC/MS) of EOCA.

The yield of recovered EOs from *Convolvulus arvensis* was 0.34% of the total mass of leaves and mainly was rich in cuprenne (34%), thymol (20%), himachalene (16%), and longifolene (10%). Most notably, EOCA was importantly comprised of sesquiterpenes hydrocarbons (66%) and oxygenated monoterpenes (22%) (Table 1 and Figure 1). The yield of recovered EOs from *Convolvulus arvensis* was 0.34%, which is comparable to that recorded in earlier work for *Convolvulus althaeoides* (0.007%) [31,32]. The chemical composition analysis showed that EOCA was found to be rich in O-Cymene, thymol, himachalene, longifolene, cuprenene, carvacrol, and cadinene (Table 1). These results are partially in accordance with earlier work reporting that *Convolvulus arvensis* possess O-cymene, carvacrol, and cadinene [19]. In addition, results are in agreement with the literature stating that the genus *Convolvulus* possesses oxygenated sesquiterpenes, sesquiterpene hydrocarbons, and oxygenated monoterpenes [31]. Notably, numerous authors researched the phytochemical composition of *C. arvensis* and found that it mostly consists of carbohydrates, coumarins, saponins, flavonoids, lipids, steroids or terpenoids, sugar derivatives of kaempferol and quercetin, tannins, alkaloids, lactones, and phenolic acids [19].

### 3.2. Antioxidant Activity of EOCA

**Figure 2:**
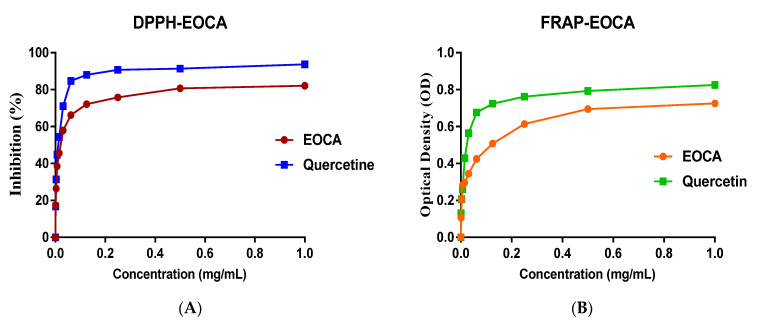
The antioxidant power of EOCA by use of the DPPH method (**A**) and FRAP method (**B**).

From the Figure 2A, it can be seen that EOCA induced important scavenging activity power. Notably, a concentration at the order of 31 µg/mL of EOAC exhibited important inhibition of free radicals, which was calculated to be 57.85 ± 2.46%, while the concentration of 500 µg/mL abolished 80.67 ± 0.68% of free radicals (Figure 2A). The analysis of the kinetics of the antiradical activity of EOCA allowed recording that EOCA inhibits free radicals in a dose–response relationship; however, this antiradical activity remains slightly less important when compared to that induced by the positive control (quercetin). The antioxidant power is frequently expressed as IC50 (half-maximal inhibitory concentration of free radicals). In this aspect, the IC50 value of EOCA was 30 µg/mL, while that of quercetin was 16 µg/mL, which are significantly different (0 ≤ 0.05)

The evaluation of antioxidant power by the use of the FRAP method (Figure 2B) revealed that EOCA exhibited antioxidant power in a dose–response relationship; importantly, the optical density (OD) increases with increasing concentrations of EOCA. Importantly, a concentration of 15 µg/mL of EOCA recorded an optical density of 0.297 ± 0.00, while a concentration of 0.250 µg/mL registered an optical density of 0.614 ± 0.008. Notably, the half-maximal effective concentration (EC50) of EOCA and quercetin were 120 µg/mL and 264 µg/mL, respectively, which were significantly different (0 ≤ 0.05)

**Figure 3:**
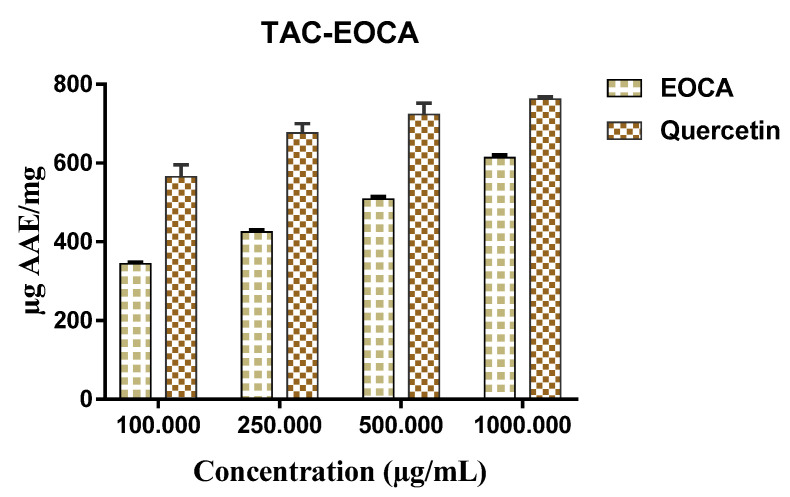
Total antioxidant activity of EOCA by use of the TAC assay.

The phosphomolybdate method used in determining the total antioxidant capacity revealed also a dose-dependent response (Figure 3). Notably, 100 µg/mL of EOCA showed a total antioxidant capacity of 344.33 ± 4.04 µg/mg, while 500 µg/mL revealed an antioxidant capacity of 508.0 ± 6.0 µg/mL. Ascorbic acid used as a reference standard showed a total antioxidant capacity of 564.67 ± 30.92 µg/mL.

The antioxidant power of EOCA determined by the use of DPPH, FRAP, and TAC assays can be referred to by the richness of EOCA in antioxidant agents, notably thymol, and carvacrol, which are recognized by their antioxidant power as reported in previous work [33]. It has been reported that antioxidant activity is related to the substitution of hydroxyl groups in phenolic aromatic rings, which contributes to the ability of EOs to hydrogenate oxygenated monoterpenes and the mixture of mono- and sesquiterpene hydrocarbons [34,35]. This suggests that the molecular structure of EOs may be responsible for their antioxidant activity. More than that, Bajalan [36], claims that the main components in EOs have a favorable correlation with their antioxidant activities. However, small chemicals have the potential to mix biological activity by direct interaction. The oxygenated monoterpene concentration of EOCA likely accounts for its antioxidant action, since this component offers a larger ability to scavenge free radicals and a superior reducing power [37,38,39,40]. Unsaturated terpenes (o-cymen, terpinene, thymol…), monocyclic monoterpenes capable of inhibiting free radicals and lipid peroxidation (which prevents cellular damage by lowering blood pressure and the cardiovascular stress response), and other monoterpenes with hydroxyl substitutes (such as terpinene and linalool) all contribute to EO’s potent antioxidant activity [41,42,43,44]. The antioxidant results shown in the work are in line with findings of a previous study, wherein it was reported that antioxidant activities of *Convolvulus althaeoides* L. leave extracts determined by DPPH assay, revealing IC50 ranging from 0.1369 ± 0.0272 mg g^−1^ to 0.432 ± 0.0018 mg g^−1^ [45]. Moreover, the current findings of antioxidant activity agreed with those reported by Nam et al., who reported that *Convolvulus arvensis* extracts possessed a powerful antioxidant effect by reducing lipid oxidation REF [46,47,48].

### 3.3. Antibacterial Activity

**Figure 4:**
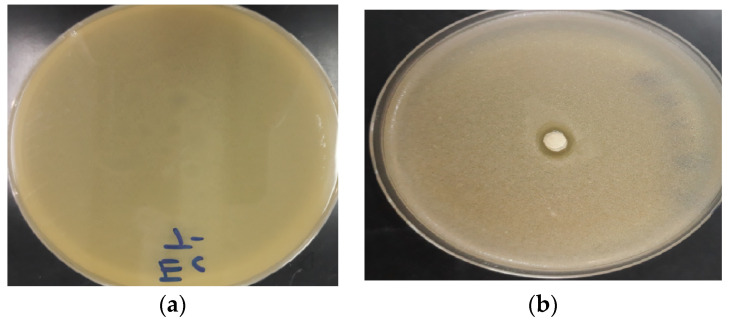
Photograph showing inhibition zones of EOCA against bacteria: (**a**) Negative control; (**b**) *K. pneumoniae*.

EOCA showed considerable antibacterial action against various bacterial species used in the experiment such as *S. pneumoniae*, *K. pneumoniae*, *E. coli*, and *S. aureus* (Table 2 and Figure 4). EOCA generated zones of inhibition against *E. coli* and *S. aureus* that were relatively large, with diameters of 19.41 ± 0.51 and 21.35 ± 0.76 mm, respectively. Notably, the antibacterial activity demonstrated against *K. pneumoniae* and *S. pneumonia* were moderate when compared to that recorded against *E. coli* and *S. aureus.* Antibacterial activity was significantly higher (*p* < 0.05) when compared to commercial antibiotics. Positive controls consisting of kanamycin and oxacillin were effective against bacteria used for testing. The MIC values for EOCA against bacterial strains ranged from 28.62 ± 1.65 to 41.27 ± 2.91 µg/mL, which was outstanding in comparison to the MICs of kanamycin and oxacillin.

The antibacterial activity of *Convolvulus arvensis* EO was tested against clinically important pathogens such as *E. coli, K. pneumoniae*, *S. pneumoniae*, and *S. aureus*, while results showed that EOCA possesses an important antibacterial effect against all strains. These results can be explained by the richness of EOCA in bioactive compounds recognized by their antibacterial properties such as thymol, carvacrol, and O-Cymene [31,32]. The obtained results in this work were comparable to those reported elsewhere [49,50,51,52]. There, it was reported that the ethanol extract of *Convolvulus arvensis* was evaluated against *Staphylococcus aureus*, *Streptococcus pyogenes*, *Escherichia coli*, and *Klebsiella pneumonia* and it showed important inhibitory effects at five various doses (500, 250, 125, 0.06, and 0.03 mg/mL). Notably, the literature reported that the leaves and stems extracts of *Convolvulus arvensis* obtained by use of butanol, chloroform, and acetone exhibited antibiotic effects against *S. aureus*, *S. epidermidis*, *S. saprophyticus*, *A. subtilis*, *A. junii*, *K. baumannii*, *E*. *aerogenes*, *E. pneumoniae*, *E. coli*, *E. faecalis*, *P. aeruginosa*, *P. vulgaris*, *S. dysenteriae*, *V. cholera*, *P. mirabilis*, *S. Typhimurium*, *S. paratyphi*, *S. marcescens*, *S. pyogenes*, and *E. cloacae* [20,33,34,35].

### 3.4. Antifungal Activity

**Figure 5:**
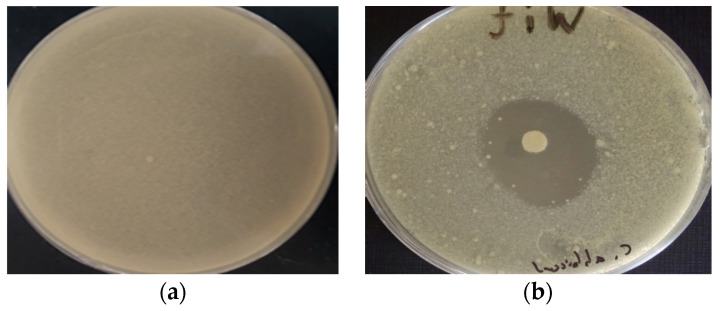
Photograph showing inhibition zones of EOCA against fungi: (**a**) Negative control; (**b**) *A. flavus*.

Important antifungal activity was shown by EOCA against all fungal strains wherein the inhibition zone diameters against all fungal species ranged from 19.44 ± 1.10 to 20.41 ± 1.81 mm (Table 3 and Figure 5). The efficacy of EOCA as an antifungal agent was comparable to that of the fungicide fluconazole, which has a powerful antifungal activity with important inhibition zone diameter versus different types of fungus. Notably, the minimum inhibitory concentrations (MICs) of EOCA against *F. oxysporum* and *C. albicans* were 18.65 ± 0.94 and 19.38 ± 0.58 g/mL, respectively. These results were significantly important in comparison to the results recorded for *A. niger* (29.39 ± 0.87) and *A. flavus* (28.94 ± 1.75). These values were substantially greater than those of the commercial fungicide fluconazole, which exhibited moderate MIC values against all strains (Table 3).

The finding demonstrated that EOCA was active against all fungal strains wherein the inhibition zone diameters against all fungal species ranged from 19.44 ± 1.10 to 20.41 ± 1.81 mm. This effectiveness may be due to the richness of EOCA in bioactive compounds with antifungal properties, e.g., thymol, carvacrol, and cymene. These compounds have been found to possess such activities, notably, thymol and cymene [53,54]. Importantly, antifungal activity was shown by *C. arvensis* organic extracts against *A. fumigatus*, *C. albicans*, *C. tropicalis*, *G. candidum*, *M. canis*, and *T. mentagrophytes*, with MIC values ranging from 0.001–0.156 mg/mL, reported in the literature [33,37]. In addition, the antifungal results shown in the present study are in accordance with findings of a previous work, wherein it was reported that extracts with increasing polarity of *Convolvulus althaeoides* L. leaves, were active against dermatophytes (*Trichophyton rubrum*, *Trichophyton mentagrophytes*, *Microsporum canis*), with inhibiting percentages reaching 100% at 50 mg mL−1. Most notably, *Candida* spp. *Candida albicans* were strongly inhibited by *Convolvulus althaeoides* L. extracts, which is in agreement with the present results. Moreover, antifungal results investigated in this study agreed with previous works on antifungal effects of volatile fractions of *Convolvulus althaeoides* L. root [32].

## 4. Conclusions

Medicinal plants have been used in the management of diseases for thousands of years and continue to provide an inexhaustible source of natural compounds to serve society. The present work showed that essential oils from *Convolvulus arvensis* had important antioxidant and antibacterial properties, which may be due to their richness in potentially active compounds such as carvacrol and thymol. Due to their promising pharmacological effects, EOs from *Convolvulus arvensis* need further evaluation in terms of toxicity on target organisms before being used for medical applications. Moreover, further works are expected to deeply investigate the mode of action of purified compounds from the studied essential oil. 

## Figures and Tables

**Table 1:** Phytochemicals identified in EOCA by GC/MS.

P	Compound	Chemical Formula	CC	KI (Lit)	KI (cal)	A (%)	
**1**	O-Cymene	C10H14	MO.H	1026	1021	5.16	
**2**	Terpinene	C10H16	MO.H	1059	1055	1.53	
**3**	Thymol	C10H14O	MO.O	1290	1289	20.10	
**4**	Carvacrol	C10H14O	MO.O	1299	1299	2.42	
**5**	Himachalene	C15H24	ST.H	1451	1448	16.50	
**6**	Longifolene	C15H24	ST.H	1407	1404	10.21	
**7**	Funebrene	C15H24	ST.H	1414	1413	1.43	
**8**	Cuprenene	C15H24	ST.H	1505	1500	34.72	
**9**	Dehydro-ar-himachalene	C15H20	ST.H	1517	1518	2.01	
**10**	Cadinene	C15H24	ST.H	1538	1535	1.20	
**11**	Elemol acetate	C17H28O2	O	1680	1680	2.57	
**12**	Himachalene oxide	C15H24O	ST.O	1524	1522	1.12	
**13**	Atlantone	C15H22O	ST.O	1694	1691	1.01	
Monoterpenes hydrocarbons (MO.H)							6.69
Monoterpenes oxygenated (MO.O)							22.52
Sesquiterpenes hydrocarbons (ST.H)							66.07
Sesquiterpenes oxygenated (ST.O)							2.13
Others (O)							2.57
Total							99.98

KI: retention index, lit: literature, Cal: calculate, A: area, P: peaks, CC: chemical class.

**Table 2:** Inhibition zone diameters and MIC results of EOCA against various bacterial strains.

	Diameter of the Inhibition (mm)			Minimum Inhibitory Concentration (µg/mL)		
Strain	EOCA	Kan	Oxa	EOCA	Kan	Oxa
*E. coli*	19.41 ± 0.51 ^b^	11.24 ± 0.65 ^a^	12.76 ± 0.68 ^a^	34.12 ± 1.04 ^b^	34.55 ± 1.28 ^a^	29.48 ± 1.19 ^a^
*K. pneumoniae*	14.27 ± 0.42 ^a^	8.42 ± 0.15 ^a^	11.17 ± 0.44 ^a^	41.27 ± 2.9 ^d^	44.72 ± 1.25 ^b^	30.52 ± 1.13 ^a^
*S. pneumoniae*	17.49 ± 0.81 ^b^	10.55 ± 0.84 ^a^	10.42 ± 0.66 ^a^	37.41 ± 3.7 ^c^	36.41 ± 1.54 ^a^	28.67 ± 1.55 ^a^
*S. aureus*	21.35 ± 0.76 ^b^	14.22 ± 0.83 ^b^	9.37 ± 0.75 ^a^	28.62 ± 1.65 ^a^	32.45 ± 1.14 ^a^	29.39 ± 1.71 ^a^

Kan: Kanamycin, Oxa: Oxacillin, EOCA: essential oil of *Convolvulus arvensis.* Column values with the same letters do not present a significant difference (Results are in means ± SD).

**Table 3:** Inhibition zone diameters and MIC results of EOCA against various fungal strains.

	Inhibition Diameter (mm)		Minimum Inhibitory Concentration (µg/mL)	
Strains	EOCA	Flu	EOCA	Flu
*A. niger*	19.44 ± 1.10 ^a^	35.05 ± 2.04 ^b^	29.39 ± 0.87 ^b^	13.73 ± 0.48 ^a^
*C. albicans*	22.37 ± 2.30 ^b^	31.18 ± 1.42 ^a^	19.38 ± 0.58 ^a^	16.84 ± 0.95 ^b^
*A. flavus*	18.92 ± 1.64 ^a^	30.26 ± 1.73 ^a^	28.94 ± 1.75 ^b^	12.92 ± 0.85 ^a^
*F. oxysporum*	20.41 ± 1.81 ^a^	32.52 ± 1.29 ^a^	18.65 ± 0.94 ^a^	13.93 ± 0.91 ^a^

Flu: Fluconazole, EOCA: essential oil of *Convolvulus arvensis*. Column values with the same letters do not present a significant difference (Results are in means ± SD).

## Data Availability

Not applicable.
